# Supplementary figures and images for: Sacubitril/valsartan preserves kidney function in rats with cardiorenal syndrome after myocardial infarction
Source: PLoS One. 2025 Oct 24;20(10):e0335214. doi: 10.1371/journal.pone.0335214 (PMC12551817; doi:10.1371/journal.pone.0335214)

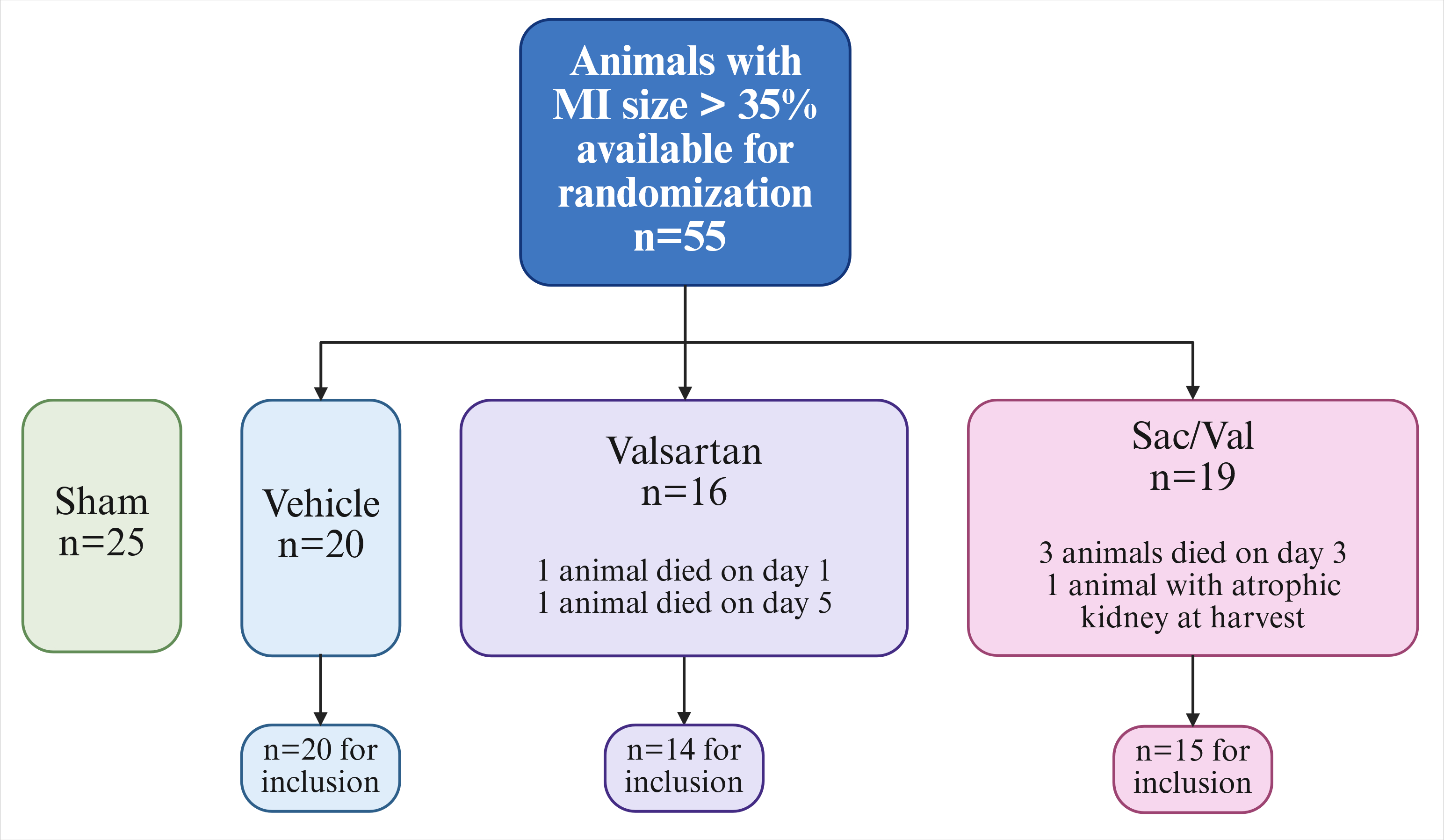

Supplement: S1 Fig — Note. 3 animals included in the analyses in the Sac/Val group were either found dead in their cage or died during MRI after 5.5–6.0 weeks. Created in BioRender. Bergo, K. (2026) https://BioRender.com/r84aurf. (TIF) [file pone.0335214.s001.tif]

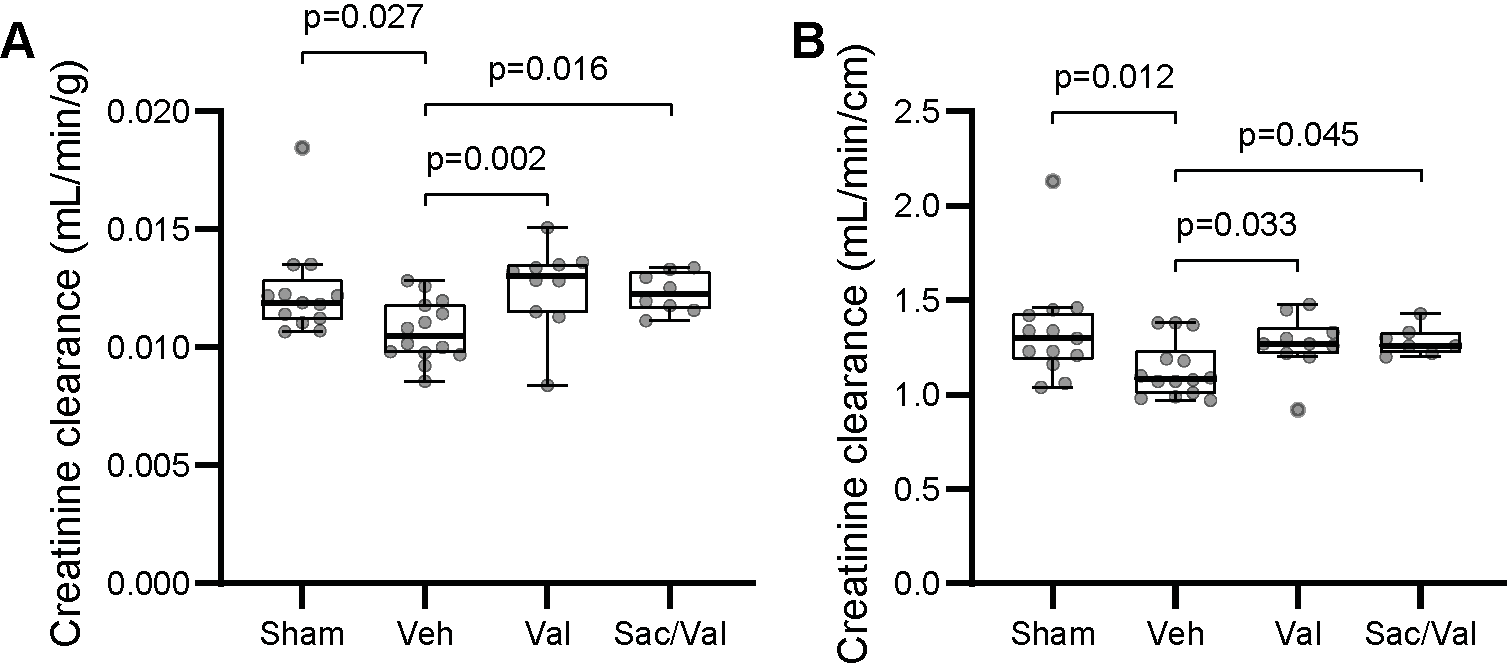

Supplement: S2 Fig — Note. Creatinine clearance normalized to Panel A: Body weight in grams and Panel B: tibia length at harvest in cm (median and IQR, Kruskal Wallis with subsequent Dunn’s test). Overall p = 0.008 for creatinine clearance/body weight and p = 0.040 for creatinine clearance/tibia length. Sac/Val = Sacubitril/valsartan, Val = Valsartan, Veh = Vehicle. (TIF) [file pone.0335214.s002.tif]
