# Supplementary material for: Sacubitril/valsartan preserves kidney function in rats with cardiorenal syndrome after myocardial infarction
Source: PLoS One. 2025 Oct 24;20(10):e0335214. doi: 10.1371/journal.pone.0335214 (PMC12551817; doi:10.1371/journal.pone.0335214)
Supplement: Attachment — Submitted filename: Response to Reviewers.docx [file pone.0335214.s004.docx]

**Comments to the Author**

1. Is the manuscript technically sound, and do the data support the conclusions?

The manuscript must describe a technically sound piece of scientific research with data that supports the conclusions. Experiments must have been conducted rigorously, with appropriate controls, replication, and sample sizes. The conclusions must be drawn appropriately based on the data presented.

Reviewer #1: Yes

Reviewer #2: Partly

2. Has the statistical analysis been performed appropriately and rigorously?

Reviewer #1: Yes

Reviewer #2: No

3. Have the authors made all data underlying the findings in their manuscript fully available?

The [PLOS Data policy](http://track.editorialmanager.com/CL0/http:%2F%2Fwww.plosone.org%2Fstatic%2Fpolicies.action%23sharing/1/010f0197c6a3cd93-a53df0e2-85ca-453f-b2b8-0f9bc433611e-000000/4XTGydnW_gMM1ZkGGGt8AT5yIQSqOrAh8772RHI6ATQ=217) requires authors to make all data underlying the findings described in their manuscript fully available without restriction, with rare exception (please refer to the Data Availability Statement in the manuscript PDF file). The data should be provided as part of the manuscript or its supporting information, or deposited to a public repository. For example, in addition to summary statistics, the data points behind means, medians and variance measures should be available. If there are restrictions on publicly sharing data—e.g. participant privacy or use of data from a third party—those must be specified.

Reviewer #1: Yes

Reviewer #2: No

4. Is the manuscript presented in an intelligible fashion and written in standard English?

PLOS ONE does not copyedit accepted manuscripts, so the language in submitted articles must be clear, correct, and unambiguous. Any typographical or grammatical errors should be corrected at revision, so please note any specific errors here.

Reviewer #1: Yes

Reviewer #2: Yes

5. Review Comments to the Author

**Reviewer #1: Comments**

The manuscript presents a clear experimental design, with a logical progression from rationale to methods and outcomes. Inclusion of relevant physiological parameters (creatinine clearance, MAP, cardiac imaging) provides a comprehensive picture of both renal and cardiac effects. The use of a post-MI rat model is clinically relevant and strengthens the translational value.

Points for Improvement

Abstract
The current title (“Sacubitril/valsartan preserves kidney function in rats after myocardial infarction”) does not fully capture the study's scope written in the abstract. Since the model involves post-MI systolic dysfunction (i.e., heart failure) and evaluates cardiorenal outcomes, the title should be revised to reflect this more accurately. Eg Sacubitril/valsartan preserves renal function in a rat model of post-MI heart failure or something like Cardiorenal protective effects of Sacubitril/valsartan after myocardial infarction in rats. Unlike just writing as after myocardial infarction

**We have changed the title to “Sacubitril/valsartan preserves kidney function in rats with cardiorenal syndrome after myocardial infarction”.**

1. The abstract lacks essential terms which are included in the key words such as ARNI, Cardiorenal syndrome etc. Please ensure the key words are also appearing in your abstract

**We have included the term “Cardiorenal syndrome” as well as “angiotensin receptor blocker and neprilysin inhibitor/ARNI”.**

2. No mention of how Sacubitril/valsartan might exert renal protection (e.g., via RAAS-neprilysin modulation, natriuretic peptides). A single phrase would help anchor biological plausibility.

**We have included the phrase “Angiotensin receptor blocker and neprilysin inhibitors (ARNI) may preserve renal function by modulating both the renin-angiotensin-aldosterone system and the natriuretic peptide system”.**

3. The concluding sentence states that Sac/Val and valsartan “preserve renal function to the same extent,” yet only Sac/Val reduced LA diameter, a meaningful difference that’s not emphasized in the conclusion. Refine the last sentence to reflect the additional cardiovascular benefit observed with Sac/Val.

**We have changed the last sentence to “Sac/Val initiated in the acute post-MI phase preserved renal function to the same extent as valsartan alone and uniquely reduced left atrial dilatation, suggesting additional benefits beyond renoprotection in the setting of persistent systolic dysfunction”.**

4. P-values should be consistently formatted (e.g., p < 0.05, not p<.05), and consider standardizing data presentation (e.g., whether median [IQR] is needed throughout for all variables or just renal). Please include actual p-value unless it’s something like 0.000 of which you may justified to included a < sign.

**This has been changed according to the suggestions.**

Methods
The MI model is robust and well-described, and the sham group inclusion strengthens internal validity. Randomization, dosing regimens, animal handling, and humane endpoints are transparently outlined, showing high procedural quality.

Points for Improvement:
1. While deaths are explained in detail, consider briefly stating how attrition affected statistical power, and whether any imputation or exclusion strategy was pre-specified. For transparency, it would be helpful to include a flow diagram summarizing final numbers per group after exclusions.

**In the Statistical methods section, we’ve included: “The attrition of animals caused a reduction in power, and non-significant results must be interpreted with care.” as well as “Missing values were excluded case-wise”.**

**Supplemental figure 1 shows the animals available for inclusion after attrition of animals.**

2. The use of a fixed solution volume (4 mL/kg) is appropriate, but it's unclear if drug concentration adjustments were made for weight gain over the 6 weeks.

**The total dose received were adjusted according to weekly weighing, which is now specified in the Methods section (Animal model and study design part).**

3. The note that some animals were used in another study [20] is appropriate. However, please clarify whether any overlapping endpoints or data reuse occurs in this manuscript.

**This has been specified in the Animal model and study design part under Methods section as: “Our previously published article on cardiac effects included the same animals as in this study in addition to an extended subset of animals for analysis of left ventricular ejection fraction, left ventricular end-diastolic volume and left atrial diameter”.**

4. The sequence of procedures (e.g., BP measurement at weeks 2.5 and 5, followed by urine collection at 5.5 weeks, then imaging at 6 weeks) is logical but would be clearer with a brief procedural timeline diagram or summary table.

**A new Figure 1 shows the timeline of the study.**

5. It's still unclear if baseline renal and cardiac function (e.g., creatinine, EF) was confirmed to be normal before MI induction in all animals. This is essential for interpreting post-MI decline as treatment effects vs. pre-existing variability.
Were creatinine clearance values normalized to body surface area or body weight? This should be stated clearly.
The use of reference samples from two healthy animals to normalize across plates is creative, but this approach should be justified and potential batch effects discussed.

**MI induction in rats is challenging, with high intra- and post-operative mortality the first few hours-days and uncertainty regarding infarct size until MRI is performed after two days. Because this investigation focused on kidney function and outcomes, we included only animals with large infarctions (i.e. >35%), as this group was expected to develop kidney impairment during the study. This led to a high number of animals undergoing MI induction, with only a minority relevant for the renal studies after MRI demonstrating the MI size. It was unfortunately not possible logistically to perform pre-MI investigations on the animals.**

**Creatinine clearance was not normalized to body weight, and this is stated in the “MI animals developed renal dysfunction with no signs of structural renal damage” part in Results section: “Due to possible differences in fluid retention between the groups, we evaluated creatinine clearance without normalization to body weight. However, normalization of creatinine clearance to body weight or tibia length reflecting body size did not change significance levels between the animal groups (Supplemental figure S2).”
Also, we added a reference to Supplemental figure S2 in the “Drug treatment preserved renal function despite lower BP levels” part of Results section, and we added the mentioned supplemental figures to demonstrate the same results regardless of normalization.**

**We have adjusted the text regarding the reference samples from two healthy animals in the methods section on ELISAs: “To reduce the significance of potential batch effects between kits, results from samples that were run on separate plates for the same analyte were normalized to the arithmetic mean of samples from two healthy animals, also run in duplicates, that were analyzed on all plates. We also made sure that samples from each group were represented on each plate.”**

6. The text refers to subsets undergoing LV catheterization and unilateral kidney perfusion, but the exact ‘n’ per procedure is not always stated clearly. This makes it harder to track sample sizes per outcome. It would be helpful to explicitly state how missing samples (e.g., deaths) were handled in analysis, was any imputation done, or were they excluded case-wise?

**The n per group has been added for LV catheterization and unilateral kidney perfusion in the Procedures section under Sacrifice at six weeks.**

**In the Statistical methods section, we’ve included: “Missing values were excluded case-wise”.**

Statistical Methods:
1. The use of bootstrapped ANOVA and non-parametric tests is appropriate.
2. However, no mention is made of sample size justification or power calculation. Even a retrospective mention (e.g., based on effect sizes from primary endpoints) would strengthen confidence in findings.

**This has been specified in the Methods section: “****The power analysis of the major study was based on an expected change of EF with 15%, standard deviation (SD) 0.2, with power 0.8 and α = 0.05, leading to the need of 22 animals in each group.** **This was based on our group’s previous experience with the model. We did not perform a separate power analysis of this renal substudy.”**

3. Specify how multiple comparisons were addressed when testing more than one outcome (e.g., collagen, glomerular area, MAP, EF, etc.).

**We primarily used non-parametric tests, which are more conservative than parametric alternatives. We considered applying formal corrections for multiple comparisons, however, given the exploratory nature of the study, such adjustment would substantially reduce statistical power and increase the risk of overlooking potentially relevant effects. As noted in the Limitations section, the renal outcomes are regarded as hypothesis-generating analyses for future investigations. For these reasons, we did not apply additional multiple comparisons adjustments.**

Results
1. The reduction in left atrial diameter with Sac/Val is important but under-discussed in the results. A more explicit acknowledgment that only Sac/Val achieved this structural improvement would strengthen interpretation, especially since systolic function was unchanged across treatments.

**We have stated more clearly that “only Sac/Val reduced left atrial diameter” in the “Drug treatment reduced BP levels and Sac/Val reduced left atrial diameter” part of the Results section.**

2. Again, While sample sizes are noted, it would be helpful to briefly mention how missing data from deaths or excluded MRI cases were handled in the final analysis…….especially since LVEF data are available in fewer animals than others.

**This has been specified in the Statistical methods section.**

3. The statement that "no visible histological changes were observed" is too casual, consider replacing with "no overt histological changes were detected on PAS or picrosirius red staining," and acknowledge the impact of suboptimal sample quality on interpretability.

**The text was changed according to the suggested phrase.**

4. The section heading claims RAAS and ANP levels were unchanged in MI animals, but Table 3 and text clearly show increased angiotensin II and ANP with Sac/Val. This contradiction needs correction for clarity.

**For clarification, the section heading is changed to “Systemic RAAS and ANP levels were unchanged in untreated MI animals”**

5. The rise in angiotensin II with Sac/Val is expected due to reduced feedback inhibition, and elevated plasma ANP supports ARNI’s mechanism. However, the text does not clearly connect these findings to the observed clinical benefits (e.g., LA remodeling, renal protection). A sentence or two linking this more explicitly would improve cohesion.

**We have added “Increased natriuretic peptide levels may reduce both cardiac preload through their natriuretic and diuretic effects and afterload through vasodilatation, which could lead to the observed normalisation of left atrial size” in the Blood pressure and cardiac effects part of the Discussion.**

**Furthermore, we have adjusted the first paragraph of the Renal function and structure section of the Discussion: “One possible mechanism through which Sac/Val may preserve renal function in the setting of lower systemic BP and RAAS inhibition as seen in the current study, is through elevated ANP levels. ANP is one of the substrates with highest affinity for neprilysin, and has been shown to cause vasodilatation of afferent arterioles and vasoconstriction of efferent arterioles, leading to increased intraglomerular pressure and increased glomerular filtration rate. However, the valsartan group improved creatinine clearance to the same extent as Sac/Val.”**

**We have also adjusted a sentence in the Hormonal effects part of the Discussion:** ***The increased angiotensin II levels in the Sac/Val group were expected as neprilysin inhibits its degradation* and added : “, but blood pressure levels are not elevated as valsartan inhibits its receptor.”**

6. The finding that valsartan reduced kidney weight is interesting but not explained. Was this due to hemodynamic unloading, structural remodeling, or measurement variation? A line of interpretation would help.

**We have added an analysis of kidney weight in sham animals vs MI animals, and updated the Statistical methods part (“Comparison between two groups (i.e. kidney weight in sham and MI animals) was performed with independent samples t-test with bootstrap.”), the Drug treatment preserved renal function despite lower BP levels section in Results (“The observed reduced kidney weight was also observed when all the MI animals were compared to the sham animals (sham animals mean weight 1.34±0.16 g and MI animals 1.22±0.11 g, p = 0.020”), as well as Renal function and structure section in Discussion (“The animals included in this study were young and still growing at the time of MI induction. The observed reduced kidney weight in MI animals is probably caused by lack of kidney growth during development due to sickness”).**

7. It is worth emphasizing that only Sac/Val improved LA diameter despite similar effects on BP and EF……..this supports a distinct hemodynamic or structural benefit and should be highlighted more assertively.

**We adjusted a sentence in Blood pressure and cardiac effects section of the Discussion: “Although global systolic dysfunction did not change with treatment in the current study, only the Sac/Val group reduced left atrial dilatation despite similar effects on BP and ejection fraction in the valsartan group”**

Discussion/conclusion/limitation
1. Refer to comment on LA diameter. Please reconcile or acknowledge this discrepancy to clarify whether Sac/Val confers any additive benefit.

**See responses to Results comment #1, 5 and 7 above.**

2. The text attributes non-significant cardiac differences to being underpowered. It would help to quantify or cite effect sizes, or mention this limitation earlier under methods or results.

**We have moved the relevant paragraph to the Results section (under Drug treatment reduced BP levels and Sac/Val reduced left atrial diameter).**

3. While plausible mechanisms for preserved renal function are discussed (e.g., afferent/efferent tone, ANP), the explanation is speculative. The discussion could benefit from highlighting the lack of structural damage despite functional decline as a unique model trait worth exploring further.

**We have adjusted the last part of the section Renal function and structure in Discussion: “This lack of structural renal damage despite functional decline might be due to the shorter observation time of our study compared to previous reports and is a unique model trait worth exploring further.”**

4. The limitations are appropriate but could be more sharply focused on missing functional readouts (e.g., no GFR, venous congestion), rather than repeating methods (light microscopy only).

**We have removed the sentence regarding light microscopy.**

5. E.g., "These protective effects occurred despite reduced systemic BP..." is restated multiple times across the text. Consider condensing to preserve flow and word economy.

**We have adjusted this.**

**Reviewer #2: Reviewer Comments:**
1. Inclusion of Histological Evidence:
There is a need to include representative histological slides in the study to support the reported findings and enhance the interpretability of the results.

**We have adjusted Figures 3 and 4 (previously 2 and 3), so that Figure 4 now includes representative images of histological slides.**

2. Abstract Revision:
The abstract should be revised to adhere to the journal’s guidelines. In particular, please ensure that the Methods section of the abstract includes key statistical details, such as the statistical tests used, and any software or analytical tools employed.

**We have not found any specific abstract guidelines for the journal except for maximum 300 words and that it should explain how the study was done without methodological detail. We did also confirm this by going through the abstracts of the last 50 articles published in PLOS ONE to make sure we did not misunderstand the guidelines, of which only one mention the statistical software used, and many do not report the statistical tests in the abstract. Hence we have decided to not add these details due to limitation on number of words.**

3. Clarification of Literature Context:
The sentence stating that "few clinical studies available did not report on creatinine changes or excluded patients with severe renal dysfunction" is broadly accurate. However, you may consider softening the language to reflect a more measured tone, such as: “While some clinical studies have not reported creatinine changes or have excluded patients with severe renal dysfunction...”

**This has been changed according to the suggestion.**

4. Paragraph Structure and Readability:
For improved readability, it is recommended that overly long paragraphs—particularly in the Animal Model and Study Design section—be split into shorter, logically structured paragraphs.

**We have gone through the entire manuscript to adjust this, especially int the Animal model and study design section.**

5. Sample Size Justification:
The manuscript does not clearly describe how the sample size was determined. Please include a justification or description of the sample size calculation or rationale, including any assumptions or references used.

**See the response to Reviewer #1’s comment regarding sample size (Statistical Methods, comment #2).**

6. PLOS authors have the option to publish the peer review history of their article ([what does this mean?](https://track.editorialmanager.com/CL0/https:%2F%2Fjournals.plos.org%2Fplosone%2Fs%2Feditorial-and-peer-review-process%23loc-peer-review-history/1/010f0197c6a3cd93-a53df0e2-85ca-453f-b2b8-0f9bc433611e-000000/50z3hxGPiqWFphzgKPdXbZ4Med8vJc8vDJkiVgbq2RE=217)). If published, this will include your full peer review and any attached files.


If you choose “no”, your identity will remain anonymous but your review may still be made public.
